# Adenocarcinoma of an Unknown Primary Site: Presentation, Diagnosis, and Management

**DOI:** 10.7759/cureus.41074

**Published:** 2023-06-28

**Authors:** Jaafar Abou-Ghaida, Adya A Ali, Sheela Anasseri, Leslie Walker, Tye Barber

**Affiliations:** 1 Osteopathic Medicine, Nova Southeastern University Dr. Kiran C. Patel College of Osteopathic Medicine, Fort Lauderdale, USA; 2 Family Medicine, Broward Health Medical Center, Fort Lauderdale, USA

**Keywords:** mcca, molecular cancer classifier assay, adenocarcinoma, cup, carcinoma of unknown primary

## Abstract

Carcinoma of unknown primary (CUP) is a rare metastatic disease in which a primary tumor site cannot be identified. CUP is a diagnosis of exclusion requiring prior workup to identify a primary site. We present a case of a 64-year-old male with vague abdominal pain, a history of gastroesophageal reflux disease (GERD), gastritis, esophagitis, hepatitis C, alcoholic pancreatitis, liver hemangioma, and Warthin tumor, and family history of cancer that was found to have CUP. The diagnosis was made after an extensive workup was done including serum tumor markers, computed tomography (CT) and ultrasound (US) imaging, flow cytometry, and an array of immunohistochemistry stains positive for only cytokeratin 7.

## Introduction

Carcinoma of unknown primary (CUP) is a rare metastatic disease in which a primary tumor site is never identified. This may occur when the primary tumor is undetectable or has completely disappeared altogether, either due to eradication by a patient’s host defenses or due to the disappearance of the initial tumor after seeding [1]. CUP accounts for 2-5% of all cancers worldwide [1,2]. It is a broad classification of cancers that include adenocarcinomas (50-70%), squamous cell carcinomas (5%), and poorly differentiated carcinomas (20-30%) [1,3,4]. Sarcomas, lymphomas, and melanomas are not included within the category of CUP due to their specific pathologies [3]. CUP metastasis occurs most commonly in solid organ sites such as the lungs, pancreas, hepatobiliary tract, and kidneys [1,3]. It is generally associated with a very poor prognosis [5].

CUP is a diagnosis of exclusion, and many clinical tests are run beforehand in an attempt to identify a primary site [3]. Initially, nonspecific serum markers such as carcinoembryonic antigen (CEA), CA-125, CA 19-9, and CA 15-3 are measured. Although these markers do not reveal the primary site, they can be used to monitor a patient’s response to chemotherapy [1,5]. Other markers such as beta-human chorionic gonadotropin (β-hCG) and alpha-fetoprotein (AFP) are also tested. An elevated level of β-hCG and AFP suggests an extragonadal germ cell testicular tumor [1]. Further workup of cancer markers includes immunohistochemistry (IHC) staining and molecular cancer classifier assay (MCCA). IHC identifies specific cancer-associated antigens such as cytokeratin 7 (CK7), cytokeratin 20 (CK20), thyroid transcription factor-1 (TTF-1), and caudal-type homeobox transcription factor 2 (CDX-2) to narrow the diagnostic possibilities [5]. Treatment for CUP tends to be empiric due to the obscure nature of the metastatic tumors. Management of CUP is done via an empiric chemotherapy regimen consisting of a platinum agent, such as cisplatin or carboplatin, and a taxane, gemcitabine, or irinotecan [5,6]. These regimens are estimated to induce responses in 25-45% of patients [5].

## Case presentation

Our patient is a 64-year-old male with a past medical history of gastroesophageal reflux disease (GERD), gastritis, esophagitis, hepatitis C, alcoholic pancreatitis, liver hemangioma, and Warthin tumor, who presented to the Emergency Department (ED) complaining of six weeks of worsening abdominal pain. His pain originated in the epigastric region and radiated bilaterally to the left and right hypochondriac regions of his abdomen. He also admitted to a 25-pound unintentional weight loss within the last six months. The patient denied any other clinical symptoms. Of note, his family history was relevant for colon cancer with metastasis to spinal cancer in his brother and melanoma in his grandmother. In addition, the patient works as a car mechanic. During the physical examination, abdominal pain was elicited upon palpation of the upper abdominal quadrants bilaterally. The rest of the examination was negative for jaundice, scleral icterus, abdominal distension or mass, hepatosplenomegaly, and Murphy’s sign. Head, eyes, ears, nose, throat, cardiovascular, pulmonary, skin, extremities, and neurological examinations were unremarkable. 

**Figure 1:**
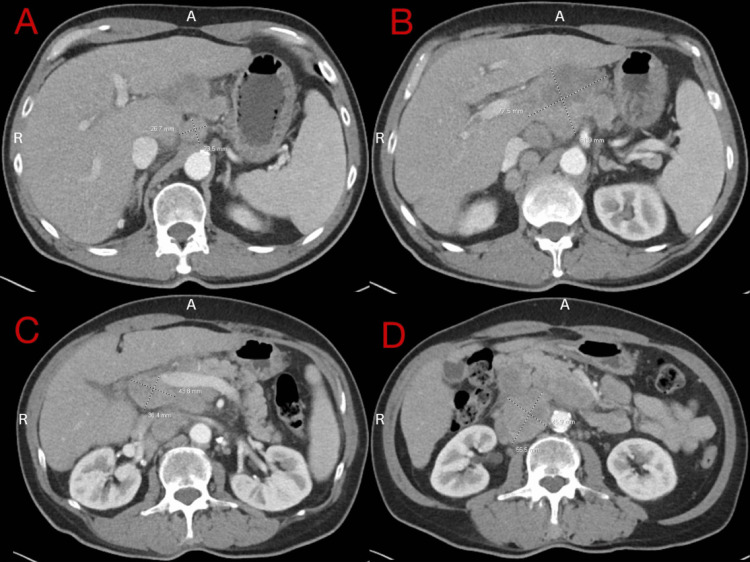
Abdominal CT revealing 26.7 x 23.5mm (A) and 77.5 x 61.9mm gastrohepatic lymph nodes with associated splenomegaly (B), and additional 36.4 x 43.8mm porta hepatis lymph node (C) and 55.5 x 45.9mm retroperitoneal para-aortic lymph node (D). A: anterior, R: right

**Figure 2:**
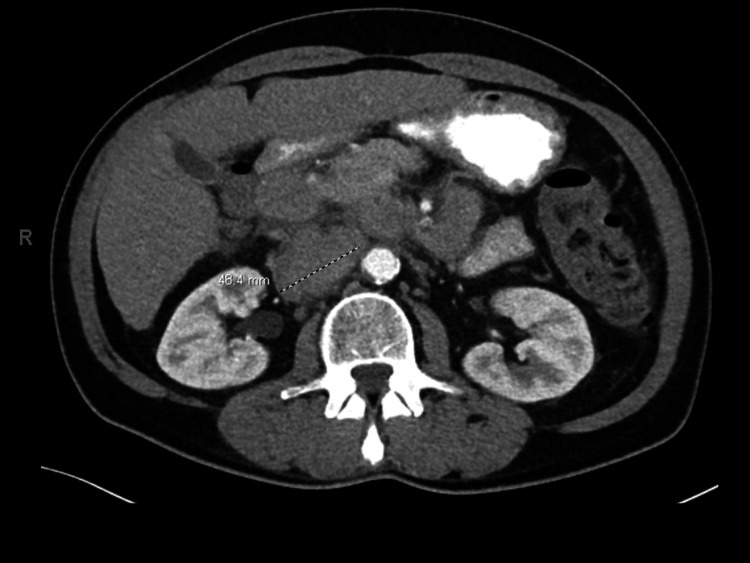
CT scan done in previous workup revealing 5cm large soft tissue attenuation suspicious of malignancy.

**Figure 3:**
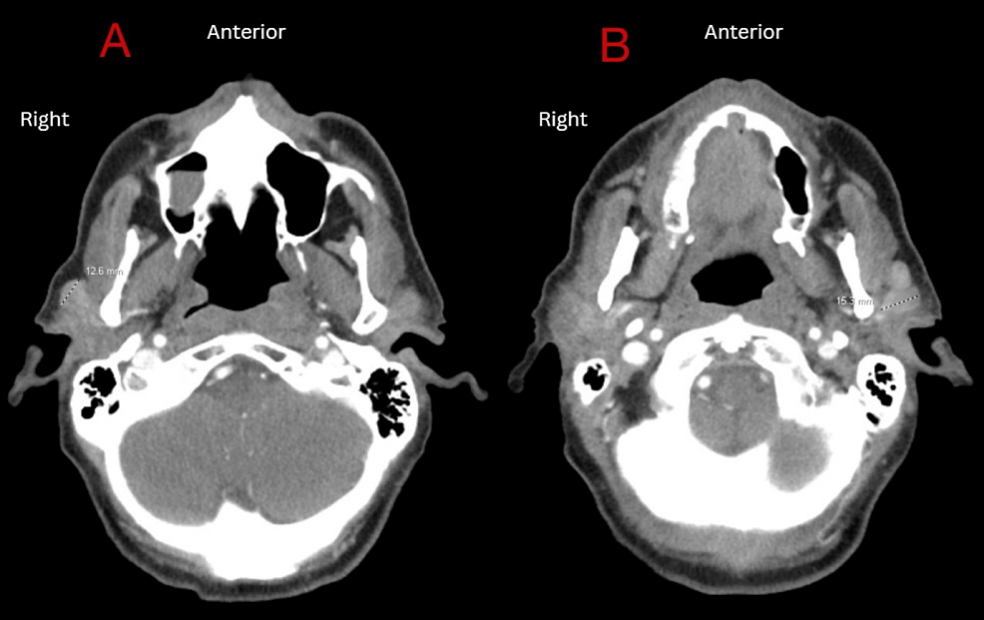
Head CT revealing a right 12.6mm (A) and a left 15.3mm (B) lymph node concerning lymphoma.

Earlier that year, the patient underwent a screening colonoscopy, which revealed early diverticulosis and tubular adenomatous polyps in the rectosigmoid area. There was no evidence of high-grade dysplasia. Upon admission, a computed tomography (CT) of the abdomen and pelvis with contrast revealed extensive mesenteric and retroperitoneal lymphadenopathy as well as mild splenomegaly (Figure 1). A comparison to CT imaging performed a few months prior due to the same vague abdominal pain revealed suspicious lymphadenopathy but to a lesser degree (Figure 2). The reason why the patient did not present earlier for evaluation is unclear. Due to underlying suspicion of lymphoma, a CT of the neck with contrast was performed, which demonstrated bilateral facial adenopathy (Figure 3). In addition, a CT scan of the chest was done; however, it did not reveal hilar lymph node enlargement or solitary lung nodules. A CT liver triple-phase showed minimal nodular contours of hepatic parenchyma. A comprehensive metabolic panel (CMP) was conducted as part of the laboratory workup, revealing a normal calcium level of 10.1 mg/dL (normal range: 8.5-10.2 mg/dL), but an elevated protein level of 10.8 g/dL (normal range: 6-8.3 g/dL). A complete blood count (CBC) was unremarkable. Lipase levels were within normal limits. Further laboratory investigations were positive for hepatitis C antibody with hepatitis C viral (HCV) load less than 15 IU/mL and HCV Log10 less than 1.18 log IU/mL (normal: 1.0-8.0 log IU/mL).

**Figure 4:**
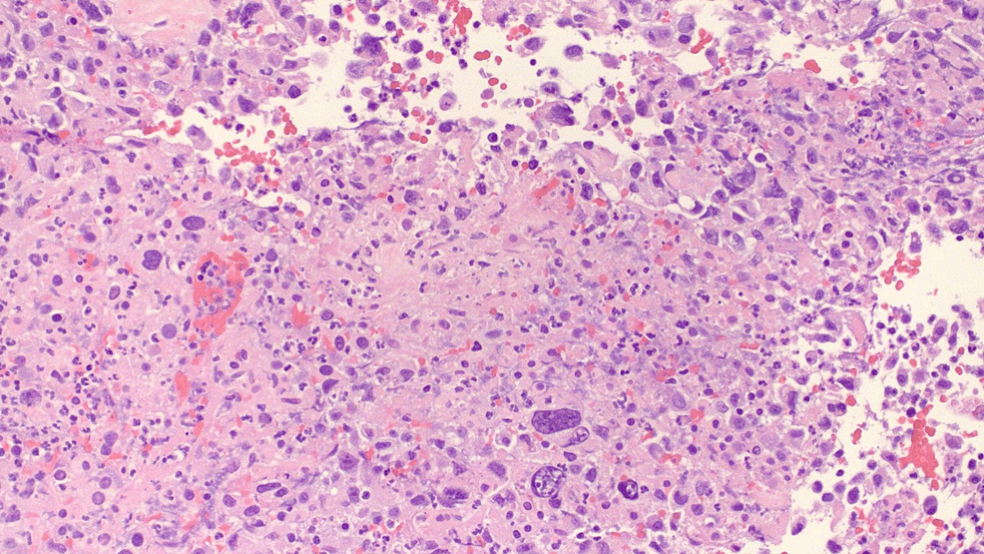
H&E staining of gastrohepatic lymph nodes presenting signs of undifferentiated carcinoma or possible metastatic pancreatic carcinoma (large pleomorphic nuclei, disorganized growth/active replication, increased nuclear to cytoplasmic ratio, and necrosis).

**Figure 5:**
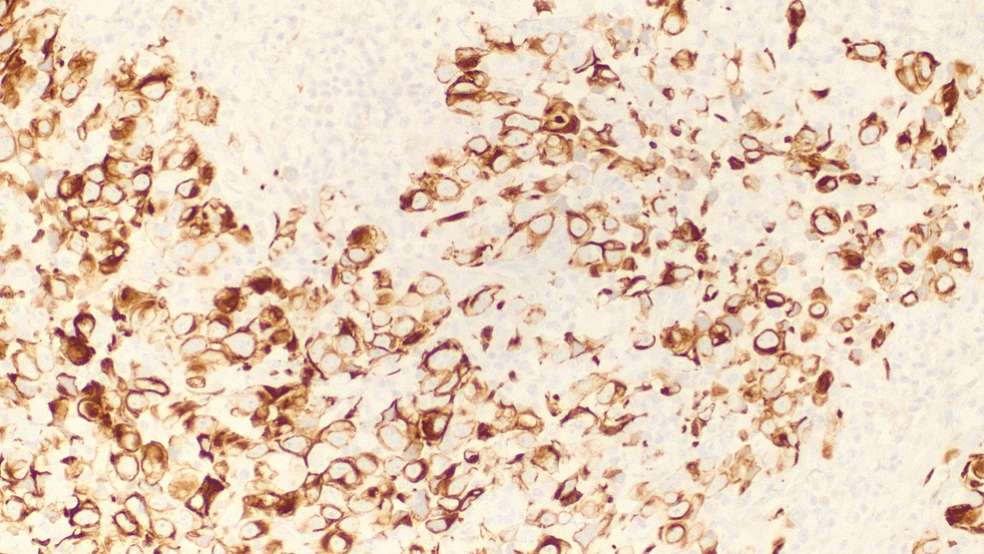
Peripancreatic tissue staining positive for CK7 on IHC. CK7, cytokeratin 7; IHC, immunohistochemistry.

Laboratory studies for cancer biomarkers revealed elevated CEA, AFP, and CA 19-9. In addition, IgG, IgA, β-2 microglobulin, free kappa light chain (Κ LC), free lambda light chain (λ LC), and the kappa/lambda light chain ratio were elevated. A bone survey did not reveal osteolytic or osteoblastic lesions. Fine-needle aspiration (FNA) of the parotid gland and neck masses were negative for carcinoma. Subsequent flow cytometry of both samples revealed no immunophenotypic markers of immune cell proliferation. Due to the elevated AFP, a scrotal ultrasound (US) was performed but did not reveal any tumors. Supraumbilical laparotomy was conducted to obtain a biopsy from the gastrohepatic and peripancreatic lymph nodes for pathological evaluation. Biopsy samples of peripancreatic tissue reveal neoplastic formation and extensive necrosis (Figure 4). IHC was only positive for CK7 (Figure 5), but negative for CK20, CDX2, S100, CD45, CD20, CD3, CD30, CD 138, AFP, TTF1, PSA, hCG, napsin A, paired-box gene 8 (PAX 8), hepatocyte antigen, arginase, placental alkaline phosphatase (pLAP), and acid-fast bacilli. The final diagnosis was then made of undifferentiated adenocarcinoma of an unknown primary site. The patient was then scheduled for a chemotherapeutic regimen consisting of paclitaxel and gemcitabine. On follow-up visits, the patient still reported bilateral abdominal pain with moderate fatigue associated with chemotherapy. Presently, the patient has completed five cycles of chemotherapy and is being evaluated by palliative care. The prognosis remains poor given the lack of a primary tumor site, generalized chemotherapy approach, and persistence of abdominal pain.

## Discussion

As mentioned before, CUP is a diagnosis of exclusion. A thorough history and physical examination followed by appropriate tests is important to rule out common etiologies while avoiding unnecessary workups. History taking should focus on prior suspicious lesions, active cancers, past surgeries, and family history of cancer [7]. In the setting of epigastric pain, blood tests and imaging studies can help rule out common abdominal conditions. IHC and flow cytometry, on the other hand, rule out common malignancies such as multiple myeloma, lymphoma, germ cell tumors, and metastatic melanomas and sarcomas. This is paramount given that such malignancies have established specific therapies that are available to treat them [7]. The following case was initially portrayed as a gastrointestinal issue due to the epigastric abdominal pain, and history of hepatitis C and pancreatitis. This necessitated a workup to rule out recurrent or new gastrointestinal pathologies. As malignancy seemed more likely given the abdominal and facial CTs and elevated serum tumor markers, common malignancies such as colon cancer, lymphoma, multiple myeloma, and germ cell tumors needed to be ruled out first.

Tumor markers such as CEA, CA-125, CA 19-9, and CA 15-3, are not specific and thus are not helpful in determining the site of the primary tumor. On the other hand, β-HCG and AFP levels have proven to be useful in evaluating extragonadal germ cell tumors [7,8]. In the following case, the patient had elevated levels of AFP, but further workup with testicular US and AFP IHC staining were negative. In addition, Κ LC, λ LC, and the kappa/lambda light chain ratio were all elevated. However, bone survey and CD138 IHC staining were negative and, as a result, not concerning for multiple myeloma. There is no clear consensus in the current literature on tumor marker reliability, with some stating lack of specificity and sensitivity and the possibility of creating emotional distress [9]. However, tumor markers can be used to determine disease progression and response to therapy.

IHC staining is superior to serum tumor markers. It can identify specific tumor subtypes, which can improve prognosis as specific treatment is instituted. For the evaluation of gastrointestinal carcinomas, IHC panels should include CK7, CK20, CDX2, and TTF1 to screen for tumors of pancreaticobiliary and gastrointestinal origins [10]. CK7+/CK20- tumors are suggestive of lung, breast, thyroid, endometrial, cervical, and pancreatic carcinoma and cholangiocarcinoma; on the other hand, CK20+/CK7- tumors are suggestive of colorectal, urothelium, and Merkel cell carcinoma [7]. In this case, IHC staining of peripancreatic lymph nodes tested positive for CK7 and negative for CK20, which indicated possible adenocarcinoma of pancreatic origins. In addition, the IHC panel tested negative for CD45, CD20, CD3, CD138, AFB, PLAP, CD30 and HCG, CK20 and CDX2, PSA, hepatocyte antigen, and arginase, ruling out lymphoma, multiple myeloma, germ cell tumors, colorectal carcinoma, prostate cancer, and hepatocellular carcinoma. Such malignancies are important considerations in this case of a male with family history of colorectal cancer, blood tests suspicious for multiple myeloma, and CT findings suspicious for lymphoma and germ cell tumors.

As stated previously, treatment of CUP remains empirical and the prognosis is poor. Regimens consisting of platinum and taxane or platinum and gemcitabine yield a response rate as low as 15-20% and a survival rate of approximately nine months [11]. In the following case, the patient still exhibited symptoms of abdominal pain despite five rounds of chemotherapy. However, advances in diagnostic modalities have allowed an increase in response rate and improvement in patient prognosis. MCCA is a promising diagnostic tool used for the identification of primary tumor sites. It allows for the sub-classification of CUP by identifying genetic alterations in specific mRNA sequences. This allows for the assessment of highly active genes within a specific cell or tissue. This assay may help not only in disease classification but also in identification of targeted treatment for patients with CUP and determination of their future prognosis [12]. Numerous studies have identified *KRAS*, *BRAF*, and *PIK3CA *as the most common genetic alterations in CUP, especially adenocarcinomas [13,14]. According to a retrospective study by Greco et. al., MCCA was successful at locating primary sites in 83% of CUP cases; subsequent initiation of targeted chemotherapy led to an increase from 25 to 72 months of survival time [15]. A recent nonrandomized clinical trial study based in Japan identified a promising increase in overall survival of 13.7 months, progression-free survival averaging 5.5 months, and a one-year survival probability in 53.1% of cases [16]. As humble as these findings are compared to Greco's, it still lends credence to the importance of primary site identification and targeted therapy initiation. Unfortunately, access to next-generation sequencing technology remains sparse, and not all CUP patients are routinely evaluated with MCCA, as was the case in this patient.

Carcinomas of unknown primary can have an insidious presentation and may prompt evaluation of solid tumors such as germ cell tumors and lymphomas, or plasma cell dyscrasias such as multiple myeloma. The following case reinforces the importance of upgrading current methods of tumor primary site detection by expanding IHC panels. MCCA and other gene expression profiling assays have shown promise in locating primary tumor locations, which may allow for the focus to be shifted away from CUP and empiric chemotherapy and toward specific cancer types and targeted therapy.

## Conclusions

In conclusion, CUP is a category of metastatic diseases in which a primary tumor site cannot be located. It is a diagnosis of exclusion requiring extensive diagnostic testing to rule out all other potential diagnoses. It reinforces the need to expand techniques to identify specific cancers and guide patient treatment appropriately. This may entail improving current IHC staining techniques to include a greater variety of cancer-associated antigens as well as further development of molecular profiling with tools like the MCCA genetic assay. Empirical chemotherapy regimens are initiated due to the unknown nature of the tumorous growths. However, with more research aimed at expanding current IHC panels and MCCAs, it may become possible to initiate targeted chemotherapy and reduce instances of CUP diagnosis as a result.
